# Algorithmic management and employee innovation: unveiling the mediating mechanisms of perceived fit, perceived control and job crafting

**DOI:** 10.3389/fpsyg.2026.1832770

**Published:** 2026-05-21

**Authors:** Rustem Korabayev, Zhen Fan, Ling Peng

**Affiliations:** 1 School of Management, Guangdong University of Science and Technology, Dongguan, China; 2 Faculty of Economics and Business, Hokkaido University, Sapporo, Japan

**Keywords:** algorithmic management, innovation performance, job crafting, perceived control, perceived fit

## Abstract

With the rapid development of digital technologies, algorithmic management has become an important management model in modern organizations. Drawing on the Job Demands-Resources theory and Self-Determination Theory, this study constructs a chain mediating model to explore how algorithmic management influences employee innovation performance through perceived fit, perceived control, and job crafting. Based on a survey and empirical analysis of 353 employees in high-technology enterprises, the results show that algorithmic management has positive effects on both perceived fit and perceived control, but the effect on perceived fit is significantly stronger than that on perceived control. Moreover, perceived fit and perceived control mediate the relationship between algorithmic management and innovation performance, with job crafting serving as a chain mediator in both pathways. Notably, the mediating effect of perceived control is very weak. This study reveals that the resource-driven mechanism of algorithmic management is significantly stronger than its demand-constraint mechanism. These findings provide theoretical insights and practical implications for organizations to better leverage algorithmic management to foster employee innovation.

## 1 Introduction

With the rapid diffusion of digital technologies, algorithmic management has emerged as a dominant mode of organizational control, initially in platform companies and increasingly in high-technology enterprises (Makó et al., 2025). Algorithmic management refers to a set of management practices in which organizations utilize data-driven algorithmic systems to assist or replace human managers in the automated allocation of work tasks, behavioral guidance, performance evaluation, and feedback regulation (Edwards et al., 2024), thereby creating a highly structured, rule-driven, and data-intensive work environment.

However, existing research presents divergent views on how algorithmic management affects employee behavior. Some studies emphasize its positive effects, arguing that algorithmic systems enhance employees perceived fit and stimulate proactive work behaviors (Lin et al., 2025; Zhang et al., 2025). Other studies point out that continuous monitoring and rule constraints of algorithmic systems increase employees perceived control, thereby weakening their autonomy (Liu and Yin, 2024; Wang et al., 2024). Nevertheless, recent studies have found that perceived control may also generate positive effects by promoting promotion-focused job crafting (Zhu et al., 2024). These divergent findings suggest that the impact of algorithmic management depends on whether employees interpret it as an enabling tool or a constraining mechanism (Liu et al., 2025; Meijerink and Bondarouk, 2023).

According to the Job Demands-Resources theory and Self-Determination Theory, the psychological perceptions triggered by algorithmic management constitute novel job demands and resources (Liang et al., 2025). When the external environment reaches employees’ psychological need thresholds, individuals actively seek behavioral adjustments to restore balance—behavioral responses that are conceptualized as job crafting. This provides a theoretical foundation for understanding perceived fit and perceived control as dual mediating mechanisms.

Current research on algorithmic management remains at an early stage, with most studies focusing on conceptual, ethical, or operational issues (Li et al., 2025; Sadeghi, 2024). Few studies have examined the chain transmission process from psychological perceptions (perceived fit and perceived control) to adaptive behaviors (job crafting) and ultimately to innovative outcomes (Jadad-Garcia and Jadad, 2024; Wang et al., 2025). Particularly in high-technology enterprises, where algorithmic systems are deeply embedded in daily operations, investigating the mechanisms through which algorithmic management affects employee innovation performance holds significant theoretical and practical value (Makó et al., 2025).

Therefore, drawing on the Job Demands-Resources theory and Self-Determination Theory, this study constructs a dual-chain mediating model to reveal how algorithmic management influences employee innovative behavior through perceived fit and perceived control (dual psychological perceptions) as well as job crafting (adaptive behavior). By uncovering this multiple transmission pathway of psychological and behavioral processes, this study aims to provide a more nuanced explanatory framework for both theoretical development and practical intervention in the field of algorithmic management.

## 2 Literature review and hypotheses development

### 2.1 Algorithmic management, perceived fit and perceived control

Algorithmic management initially emerged in the platform economy (e.g., food delivery, ride-hailing services). The concept was first introduced by Lee et al. (2015), who found in their study of Uber and Lyft that software algorithms were taking over functions traditionally performed by human managers, including task allocation, optimization, and evaluation. Their research suggested that algorithmic management enables firms to coordinate and manage labour on a large scale through data-driven approaches in the absence of traditional employment relationships. With the rapid diffusion of digital technologies and the flourishing development of the platform economy, academic attention to this concept has steadily increased, and the research scope has gradually expanded from initial platform work contexts to broader organizational management settings. Scholars have begun to define algorithmic management more broadly as the practice of delegating managerial functions to algorithms and automated systems, or understanding it as “using artificial intelligence algorithms to control how people perform and complete work processes” (Deng et al., 2025; Wood, 2024).

Current research on algorithmic management generally recognizes that its effects on employee psychology and behavior are two-sided. On the one hand, algorithmic management can improve the fairness and efficiency of task allocation, provide employees with clear work guidance and real-time feedback, thereby enhancing employees’ clarity of task requirements (Gong, 2025); the optimization functions of algorithmic systems (e.g., route planning, intelligent task allocation) and dynamic incentive mechanisms can also improve employee work efficiency and income (Khamis, 2024). On the other hand, the continuous monitoring and rule constraints of algorithmic systems may generate psychological pressure of “being controlled,” weakening employees’ autonomy and intrinsic motivation (Milanez et al., 2025); excessive algorithmic control may also trigger employees’ threat perceptions and reduce work performance. This duality suggests that employees’ perceptions and interpretations of algorithmic systems serve as key determinants of whether algorithmic management yields positive or negative outcomes.

From the perspective of the Job Demands-Resources model, algorithmic management simultaneously encompasses both job resource and job demand attributes. The clear task guidance, real-time performance feedback, and fair task allocation provided by algorithmic systems constitute job resources, which help improve employee work efficiency and engagement. In contrast, the continuous monitoring, rule constraints, and inflexible automated decision-making of algorithmic systems constitute job demands, which continuously consume employees’ cognitive resources and psychological autonomy (Kinowska and Sienkiewicz, 2023; Sharma et al., 2026).

Therefore, this study proposes that algorithmic management is an organizational control model that employs data-driven algorithmic systems to perform traditional managerial functions (including task allocation, behavioral guidance, performance monitoring, and feedback regulation); it influences employees’ adaptive behaviors (such as job crafting) and innovation performance by shaping their perceptions of task fit and algorithmic control. The study proposes the following research hypotheses.

*H1*: Algorithmic management has a positive effect on perceived fit.

*H2*: Algorithmic management has a positive effect on perceived control.

### 2.2 Perceived fit, perceived control and job crafting

Regarding how algorithmic management affects employees’ job crafting and innovative behavior, existing research presents diverse perspectives. Some studies emphasize the positive effects of algorithmic management, arguing that algorithmic systems, by optimizing task allocation, providing real-time performance feedback, and enhancing process transparency, can improve employees’ perceived fit, thereby stimulating their willingness to proactively adjust work behaviors and promoting job crafting and innovation (Lin et al., 2025; Liu and Yin, 2024). Other studies focus on the negative effects of algorithmic management, pointing out that continuous monitoring, rule constraints, and inflexible automated decision-making of algorithmic systems increase employees’ perceived control, weaken their autonomy and intrinsic motivation, thereby inhibiting job crafting behaviors and reducing innovation performance (Wang et al., 2024, 2025).

Notably, recent research on the relationship between perceived control and job crafting has revealed certain divergences. Some empirical studies have found that perceived control may have a positive impact on job crafting. Zhu et al. (2024)found that perceived algorithmic control had a positive indirect effect on service performance through job crafting. Further research distinguished between two types of job crafting and found that perceived algorithmic control positively influenced work engagement through promotion-focused job crafting. Shi et al. (2025) also confirmed that perceived control positively influenced thriving at work through job crafting. These studies suggest that the impact of perceived control on job crafting is not unidirectionally negative but may simultaneously contain both positive and negative pathways, depending on how employees interpret and respond to algorithmic control.

Against this research background, perceived fit and perceived control have become two key psychological mechanisms for understanding how algorithmic management affects job crafting. Perceived fit refers to employees’ subjective evaluation of the degree of alignment between their personal characteristics and work environment characteristics. Employees with higher perceived fit, who believe that their job tasks and organizational environment are congruent with their personal characteristics, tend to engage in more job crafting behaviors because work conditions are experienced as supportive and meaningful (Kidron and Rispler, 2025). Perceived control refers to the extent to which employees perceive that their behaviors are being monitored, constrained, and directed by algorithmic systems (Liang et al., 2025).

In algorithmic management systems, when employees experience a strong sense of fit with algorithmic task structures, they are more likely to craft their work in ways that support adaptation and innovation (Deng et al., 2026). At the same time, algorithmic systems control behavioral expectations, which may lead different employees to produce different behavioral outcomes. For example, employees with high cognitive abilities tend to perceive algorithmic control as a predictable rule framework, thereby actively seeking optimization space within algorithmically defined boundaries by adjusting task execution sequences, integrating additional information resources, or restructuring problem-solving strategies to engage in promotion-focused job crafting. In contrast, employees with low cognitive abilities may perceive algorithmic control as an insurmountable constraint, making them more likely to exhibit compliance-oriented passive behaviors or even work withdrawal. Such individual differences lead perceived fit and perceived control to potentially have different directions of influence on job crafting.

Based on the above theoretical discussion of perceived fit, perceived control, and job crafting, this study proposes the following hypotheses.

*H3*: Perceived fit has a positive effect on job crafting.

*H4*: Perceived control has a positive effect on job crafting.

### 2.3 Perceived fit, perceived control, job crafting and employee innovation performance

Employee innovation performance refers to employees’ discretionary and proactive behaviors aimed at generating, advocating, and implementing new ideas to improve products, processes, or services (Scott and Bruce, 1994). As observable behavioral outcomes, innovation-related behaviors depend not only on individual capabilities and intrinsic motivation but also on enabling contextual factors, such as autonomy, supportive organizational culture, perceived organizational support, and transparent management practices, which allow employees to experiment, take risks, and apply creative solutions (Law and Varanasi, 2025; Lee and Kim, 2024).

When employees proactively adjust task boundaries (e.g., changing work methods, redesigning processes), relational boundaries (e.g., seeking colleague feedback, expanding collaboration networks), or cognitive boundaries (e.g., reframing work meaning), they are essentially creating more favorable conditions for creative problem-solving (Wrzesniewski and Dutton, 2001). Through job crafting, employees can more effectively match their personal strengths with work demands, thereby releasing more cognitive resources for innovative activities.

Research indicates that the influence of job crafting on innovation performance can be realized through multiple pathways. First, task crafting enables employees to adjust work methods according to their expertise, thereby solving complex problems more effectively and generating novel solutions (Cai and Ali, 2024). Second, relational crafting helps employees build broader social networks, facilitating knowledge sharing and cross-disciplinary collaboration, which provides social resources for the generation and implementation of innovative ideas (Ramadhani et al., 2025). In addition, cognitive crafting enhances employees’ intrinsic motivation and work engagement by reframing the meaning and value of work, and intrinsic motivation is a key factor driving creative behavior (Deng et al., 2026).

In the context of algorithmic management, the standardization and routinization tendencies of algorithmic systems may limit employees’ space for free exploration. However, through proactive job crafting, employees can seek innovation opportunities within the gaps of algorithmic constraints, such as adjusting task execution sequences, seeking additional information resources, or reinterpreting the meaning of performance feedback. Therefore, job crafting becomes a key adaptive behavior for employees to maintain and enhance innovation performance under algorithmic management environments.

Based on the above discussion, this study proposes the following hypothesis.

*H5*: Perceived fit has a positive effect on employee innovation performance.

*H6*: Perceived control has a positive effect on employee innovation performance.

*H7*: Job crafting has a positive effect on employee innovation performance.

### 2.4 The mediating role of perceived fit and perceived control

According to the Job Demands-Resources theory, perceived fit and perceived control serve as two key psychological mechanisms, representing the “resource” and “demand” attributes of algorithmic management, respectively. When algorithmic systems enhance employees perceived fit by optimizing task allocation, providing clear performance feedback, and increasing process transparency, employees interpret algorithmic structures as supportive resources. This positive psychological experience strengthens employees’ intrinsic motivation and psychological empowerment, thereby stimulating their proactive adjustments to task boundaries, relational boundaries, and cognitive boundaries in the form of job crafting, ultimately enhancing innovation performance (Keegan and Meijerink, 2025; Lippert, 2025). When algorithmic systems increase employees perceived control through continuous monitoring, rule constraints, and automated decision-making, a high level of perceived control essentially constitutes a job demand, thereby affecting employees’ intrinsic motivation and willingness to engage in proactive behaviors.

From the perspective of Self-Determination Theory, perceived fit and perceived control jointly drive employees’ behavioral intentions. When employees feel that their abilities are highly aligned with task requirements, they experience a stronger sense of efficacy, and the satisfaction of this competence need is a key factor driving intrinsic motivation and proactive behavior (Ryan and Deci, 2000). Conversely, when employees perceive that their behaviors are highly monitored and constrained by algorithmic systems, their autonomy need is threatened, their intrinsic motivation is subsequently weakened, thereby reducing their tendency to engage in proactive behavioral adjustments.

Researchers have also confirmed the mediating role of perceived fit and perceived control. Zayid et al. (2024)found that person-job fit significantly moderated the relationship between algorithmic management practices and job burnout as well as perceived threat, thereby affecting employee well-being. Zhu et al. (2024)demonstrated that perceived control had a positive indirect effect on service performance through job crafting. Furthermore, Jing and Wei (2025)found that algorithmic management has an inverted U-shaped effect on creativity, with intrinsic motivation playing a mediating role, and job crafting alleviating this nonlinear relationship. Therefore, perceived fit and perceived control play important mediating roles between algorithmic management, job crafting, and innovation performance.

Based on the above discussion, this study proposes the following mediating hypotheses.

*H8*: Perceived fit mediates the relationship between algorithmic management and employee innovation performance.

*H9*: Perceived control mediates the relationship between algorithmic management and employee innovation performance.

### 2.5 The chain mediating effects of perceived fit, perceived control, and job crafting

Regarding the resource-driven pathway of algorithmic management, existing research has shown that perceived fit is an important antecedent for stimulating employees’ proactive behaviors. When employees perceive that algorithmically assigned tasks are highly aligned with their abilities and preferences, they interpret algorithmic systems as supportive tools, thereby generating higher levels of work engagement and proactive behavioral intentions (Keegan and Meijerink, 2025). This positive psychological state further motivates employees to engage in job crafting behaviors, including adjusting task execution methods, seeking additional resources, and reframing work meaning (Demerouti, 2026). Through job crafting, employees can more effectively match their personal strengths with work demands, thereby releasing cognitive resources for creative problem-solving and ultimately enhancing innovation performance (Cai and Ali, 2024). Perceived control exhibits a different mechanism in its influence on employee behavior. Research has found that continuous monitoring and rule constraints of algorithmic systems increase employees perceived control, and a high level of perceived control essentially constitutes a consumptive job demand.

In recent years, scholars have begun to focus on the integrated study of these two chain pathways. Ali and Ishaq (2025)found that perceived control had a positive indirect effect on service performance through job crafting, suggesting that perceived control may also generate positive effects by stimulating promotion-focused job crafting. Zhu et al. (2025) further distinguished between two types of job crafting and found that perceived control positively influenced work engagement through promotion-focused job crafting while simultaneously generating negative effects through prevention-focused job crafting. Liu and Yin (2024) also demonstrated that algorithmic management increases promotion-focused job crafting by stimulating gamified experiences while increasing prevention-focused job crafting by inhibiting perceived work autonomy. These findings collectively suggest that the influence of perceived control on job crafting may simultaneously contain both positive and negative pathways, depending on how employees interpret and respond to algorithmic control.

From the perspective of theoretical integration, the combination of the Job Demands-Resources theory and Self-Determination Theory provides a complementary explanatory framework for understanding the chain mediating effects. The former explains how algorithmic management influences employees’ behavioral energy through the balance of “resources and demands”; the latter reveals how algorithmic management affects employees’ intrinsic motivation by satisfying or threatening basic psychological needs. The simultaneous existence of the two chain pathways reflects the double-edged sword effect of algorithmic management on employee innovation performance, with the overall effect depending on the relative strength of the resource-driven mechanism versus the demand-consumption mechanism.

Based on the above analysis, this study proposes the following chain mediation hypotheses:

*H10*: Perceived fit and job crafting play a chain mediating role in the relationship between algorithmic management and employee innovation performance.

*H11*: Perceived control and job crafting play a chain mediating role in the relationship between algorithmic management and employee innovation performance.

### 2.6 Research model

**Figure 1:**
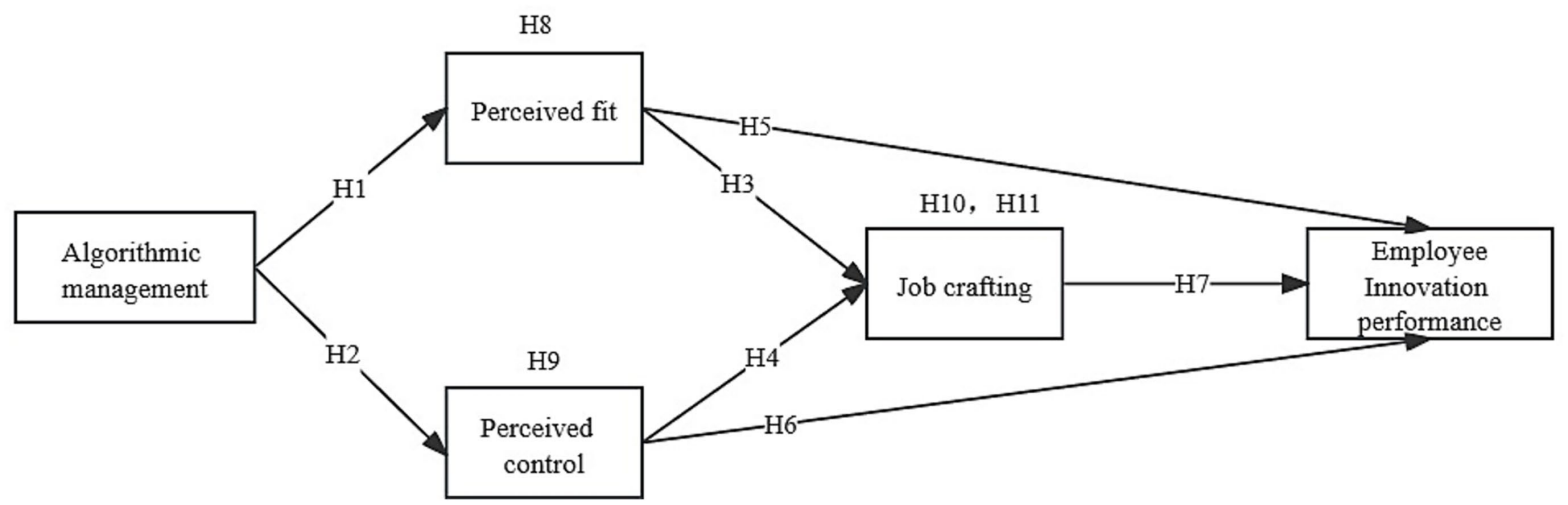
Research model.

Based on the above research hypotheses, this study constructs the following research model (Figure 1). The model includes five variables and eleven hypothesized paths, aiming to explore the relationships between algorithmic management, perceived fit, perceived control, job crafting, and employee innovation performance. The proposed model examines how algorithmic management influences employee behaviors and innovation outcomes through perceived fit and perceived control, as well as the mediating role of job crafting in this process.

## 3 Methodology

### 3.1 Data source

The data for this study were collected from employees of 50 high-technology enterprises undergoing digital transformation located in the high-tech parks of Beijing, Shanghai, and Shenzhen. The survey was conducted from May to September 2025, spanning a total of five months.

To ensure that the sample adequately represented the characteristics of employees across different subsectors, this study employed a stratified random sampling method based on the types of enterprises in the three high-tech parks. The sample covered multiple high-technology subsectors, including software and IT services, electronic equipment manufacturing, telecommunications, biotechnology, and new energy technology. A total of 80 questionnaires were distributed to each subsector, resulting in 400 questionnaires in total. In terms of geographical distribution, Beijing accounted for 35%, Shanghai for 38%, and Shenzhen for 27%, ensuring regional representativeness of the sample.

To ensure the adequacy of the sample size, this study used G-Power software to calculate the required sample size. With an effect size of 0.15 (medium effect), significance level *α* = 0.05, and statistical power 1-*β* = 0.80, the minimum required sample size was calculated to be 160. Considering the number of observed variables and the model fit requirements, the sample size was increased approximately two-fold. Therefore, a total of 400 questionnaires were distributed to employees through the online platform Questionnaire Star, with the assistance of human resources departments from each company.

To mitigate potential common method bias, the survey was administered anonymously, and items measuring different constructs were interleaved. After data screening to exclude incomplete responses, straight-lining patterns, and responses with excessively short completion times, 353 valid responses were retained, yielding an effective response rate of 88.25%, which met the sample size requirements.

### 3.2 Measurement instruments

This study employed a structured questionnaire using a five-point Likert scale (1 = strongly disagree, 5 = strongly agree). The questionnaire consisted of 28 items, with each scale adapted from validated measures in previous research (see Table 1). The reliability of each scale was assessed using Cronbach’s *α* coefficient. The results showed that all α values exceeded the acceptable threshold of 0.70, indicating that the scales had good internal consistency.

**Table 1:** Constructs and reliability.

Construct	Abbreviation	No. of items	Cronbach’s *α*
Algorithmic management	AIM	6	0.902
Perceived fit	PF	5	0.847
Perceived control	PC	5	0.842
Job crafting	JC	6	0.888
Employee innovation performance	IP	6	0.898

#### 3.2.1 Algorithmic management (AIM)

Adapted from Parent-Rocheleau et al. (2024), this scale measures employees’ perceptions of algorithmic monitoring, data feedback, and automated decision-making. Sample items include, “The algorithmic system monitors my work progress and operational behaviors in real time,” “The algorithmic system automatically generates performance evaluations based on my work data,” and “The algorithmic system assigns work tasks without human intervention.” The scale demonstrated high internal consistency (Cronbach’s *α* = 0.902).

#### 3.2.2 Perceived fit (PF)

Derived from Cable and DeRue (2002), this scale assesses the congruence between employees’ skills, values, and algorithm-assigned tasks. Sample items include, “The tasks assigned by the algorithm match my skills and abilities,” “My personal abilities can well meet the task requirements of the algorithmic system,” and “The algorithm system’s performance requirements are consistent with my personal values.” The reliability of this scale was acceptable (Cronbach’s *α* = 0.847).

#### 3.2.3 Perceived control (PC)

Based on Liang et al. (2025), this scale assesses the extent to which employees feel constrained and monitored under algorithmic management. Sample items include, “Under the constraints of the algorithmic system, it is difficult for me to decide autonomously how to perform my work,” “I feel that my work behaviors are strictly controlled by the algorithmic system,” and “Overall, the algorithmic system makes me feel highly controlled and constrained.” The Cronbach’s α for this scale was 0.842.

#### 3.2.4 Job crafting (JC)

Adapted from Wrzesniewski and Dutton (2001), this scale reflects employees’ self-initiated changes in task, relational, and cognitive domains. Sample items include, “I proactively change my work methods to better complete tasks,” “I redesign work processes to improve work efficiency,” and “I reframe the meaning and value of my work to enhance intrinsic motivation.” The scale showed good internal consistency (Cronbach’s *α* = 0.888).

#### 3.2.5 Employee innovation performance (IP)

Based on Prajogo and Ahmed (2006), this scale evaluates employees’ idea generation, implementation, and technological innovation. Sample items include, “I proactively propose new ideas to improve work processes,” “I try to apply new ideas to my actual work,” and “I try unconventional solutions in my work.” The reliability of this scale was high (Cronbach’s α = 0.898).

## 4 Results and discussion

### 4.1 Descriptive statistics

Among the 353 valid responses, 59.8% of the respondents were female, and 40.2% were male. In terms of age distribution, the largest group was 26–35 years old (30.0%), followed by 18–25 years (25.8%), 36–45 years (23.5%), and 46 years or older (20.7%). Regarding education, the majority of respondents held a bachelor’s degree (54.1%), followed by those with an associate degree or lower (33.2%) and a master’s degree or higher (12.7%). In terms of work experience, most respondents had 3–5 years of experience (34.3%), followed by 6–10 years (28.0%), 11 years or more (21.5%), and 0–2 years (16.1%). Based on the descriptive statistics of the sample, the mean values range from 3.39 to 3.75, indicating that respondents have a moderate perception of the relevant variables. All skewness and kurtosis values fall within the acceptable range of ±2 (Kline, 2015), suggesting that the sample follows a normal distribution, making it suitable for multivariate analysis (Table 2).

**Table 2:** Descriptive statistics.

Variable	N	Min	Max	Mean	SD	Skewness	Kurtosis
Algorithmic management	353	1.17	4.83	3.64	0.82	−0.82	0.04
Perceived fit	353	1.33	4.83	3.44	0.77	−0.48	−0.43
Perceived control	353	1.33	4.83	3.32	0.78	−0.44	−0.49
Job crafting	353	1.17	4.83	3.39	0.87	−0.76	−0.03
Employee innovation performance	353	1.17	4.83	3.75	0.86	−1.12	0.62

### 4.2 Reliability and validity analysis

As reported in Section 3.2, the Cronbach’s α values for all constructs exceeded 0.80, indicating satisfactory internal consistency. The KMO measure of sampling adequacy was 0.916, exceeding the recommended threshold of 0.80, and Bartlett’s test of sphericity was significant (χ^2^(435) = 5596.249, *p* < 0.001), indicating that the data were suitable for factor analysis.

As presented in Table 3, Harman’s single-factor test indicated that the first factor accounted for 31.16% of the total variance, below the 50% threshold, suggesting that common method bias is not a serious concern. Furthermore, the five extracted factors with eigenvalues greater than 1 collectively explained 62.59% of the total variance, with no single factor dominating the variance explanation, providing evidence of adequate construct validity.

**Table 3:** Common method bias test.

Component	Initial eigenvalues	Extraction sums of squared loadings				
	Total	% of variance	Cumulative %	Total	% of variance	Cumulative %
1	9.349	31.163	31.163	9.349	31.163	31.163
2	3.126	10.421	41.584	3.126	10.421	41.584
3	2.411	8.038	49.622	2.411	8.038	49.622
4	2.104	7.015	56.637	2.104	7.015	56.637
5	1.787	5.957	62.594	1.787	5.957	62.594
6	0.967	3.224	65.818			

Extraction method: principal component analysis. Only the first five components with eigenvalues > 1 are retained.

As shown in Table 4, the standardized factor loadings for all retained items ranged from 0.675 to 0.843, exceeding the recommended threshold of 0.50, indicating that each item substantially loaded onto its respective construct. The composite reliability (CR) values ranged from 0.846 to 0.908, exceeding the recommended threshold of 0.70 (Fornell and Larcker, 1981), demonstrating good internal consistency across all constructs. The average variance extracted (AVE) values ranged from 0.525 to 0.621, all above the acceptable criterion of 0.50, further supporting adequate convergent validity.

**Table 4:** Convergent validity.

Construct	Item	Factor loading	CR	AVE
Algorithmic management	AIM1	0.773	0.908	0.621
	AIM2	0.771		
	AIM3	0.745		
	AIM4	0.813		
	AIM5	0.758		
	AIM6	0.812		
Perceived fit	PF1	0.725	0.847	0.528
	PF2	0.675		
	PF3	0.728		
	PF4	0.763		
	PF5	0.737		
Perceived algorithmic control	PC1	0.689	0.846	0.525
	PC2	0.710		
	PC3	0.710		
	PC4	0.727		
	PC5	0.763		
Job crafting	JC1	0.843	0.887	0.569
	JC2	0.699		
	JC3	0.740		
	JC4	0.791		
	JC5	0.703		
	JC6	0.755		
Innovation performance	IP1	0.831	0.894	0.586
	IP2	0.726		
	IP3	0.709		
	IP4	0.796		
	IP5	0.766		
	IP6	0.798		

CR = composite reliability; AVE = average variance extracted.

Discriminant validity was evaluated using the Fornell-Larcker criterion. As shown in Table 5, the square root of AVE for each construct exceeded the corresponding inter-construct correlations, providing strong evidence of adequate discriminant validity.

**Table 5:** Discriminant validity (Fornell-Larcker criterion).

Construct	AIM	PF	PC	JC	IP
AIM	**0.788**				
PF	0.425	**0.726**			
PC	0.248	0.188	**0.720**		
JC	0.411	0.445	0.218	**0.754**	
IP	0.465	0.414	0.239	0.535	**0.766**

Diagonal values (in bold) are the square roots of the average variance extracted (AVE). All correlations are significant at *p* < 0.01.

### 4.3 Structural equation model and hypothesis testing

#### 4.3.1 Model fit

**Figure 2:**
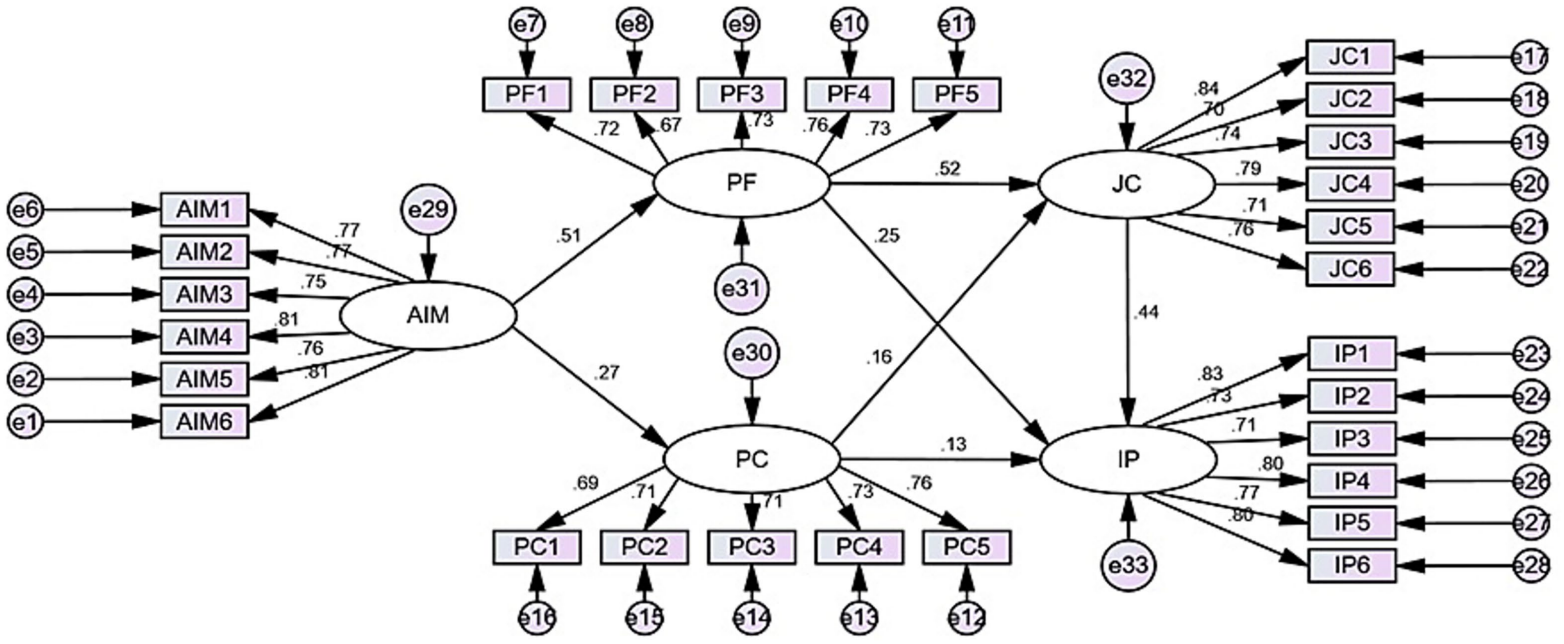
Structural equation model.

Structural equation model was conducted to test the hypothesized relationships among the study constructs (Figure 2). The model fit indices presented in Table 6 indicate a good fit of the structural model to the data. Specifically, CMIN/DF was 1.820 (<3), while GFI = 0.881, AGFI = 0.861, IFI = 0.933, TLI = 0.933, and CFI = 0.939, RMSEA = 0.048 (<0.05), all met the recommended thresholds, suggesting that the model adequately represents the observed data.

**Table 6:** Model fit indices.

Fit index	CMIN/DF	GFI	AGFI	IFI	TLI	CFI	RMSEA
Observed value	1.820	0.881	0.861	0.939	0.933	0.939	0.048
Recommended threshold	< 3	> 0.8	> 0.8	> 0.9	> 0.9	> 0.9	<0.05

#### 4.3.2 Hypothesis testing

##### 4.3.2.1 Direct effects

As shown in Figure 2 and Table 7, all hypothesized direct effects were supported. Algorithmic management positively influenced perceived fit (*β* = 0.512, *p* < 0.001) and perceived control (*β* = 0.269, *p* < 0.001), supporting H1 and H2. Perceived fit positively predicted job crafting (*β* = 0.516, *p* < 0.001) and innovation performance (*β* = 0.245, *p* < 0.001), supporting H3 and H5. Perceived control positively predicted job crafting (*β* = 0.161, *p* < 0.01) and innovation performance (*β* = 0.128, *p* < 0.05), supporting H4 and H6. Finally, job crafting had a strong positive effect on innovation performance (*β* = 0.440, *p* < 0.001), supporting H7. These findings indicate that perceived fit is a stronger driver of job crafting and innovation performance than perceived control.

**Table 7:** Standardized direct effects.

Path	Estimate	*p*-value	Support
AIM → PF	0.512	***	Supported
AIM → PC	0.269	***	Supported
PF → JC	0.516	***	Supported
PC → JC	0.161	0.003	Supported
PF → IP	0.245	***	Supported
PC → IP	0.128	0.013	Supported
JC → IP	0.440	***	Supported

AIM = algorithmic management; PF=perceived fit; PC=perceived control; JC = job crafting; IP=innovation performance;*** mean *p* < 0.001.

##### 4.3.2.2 Indirect effects

The standardized indirect effects are presented in Table 8. Bootstrap analysis with 5,000 resamples was conducted to test the mediating and chain mediating effects.

**Table 8:** Standardized indirect effects.

Path	Estimate	95% CI	*p*
AIM → PF → IP	0.125	[0.012, 0.176]	< 0.001
AIM → PC → IP	0.036	[0.008, 0.072]	0.002
AIM → PF → JC → IP	0.115	[0.055, 0.151]	< 0.001
AIM → PC → JC → IP	0.02	[0.005, 0.068]	0.027
AIM → IP (Total indirect effect)	0.296	[0.095, 0.198]	< 0.001

Bootstrap 5,000 resamples. All indirect effects are significant as the 95% confidence intervals do not contain zero.

The indirect effect of algorithmic management on innovation performance through perceived fit was significant (*β* = 0.125, 95% CI [0.012, 0.176], *p* < 0.001), supporting H8. The indirect effect through perceived control was also significant, albeit smaller (*β* = 0.036, 95% CI [0.008, 0.072], *p* = 0.002), supporting H9.

The chain mediating effects were both significant. The indirect effect through perceived fit and job crafting was *β* = 0.115 (95% CI [0.055, 0.151], *p* < 0.001), supporting H10, while the indirect effect through perceived control and job crafting was *β* = 0.020 (95% CI [0.005, 0.068], *p* = 0.027), supporting H11.

These results indicate that perceived fit and perceived control serve as mediators between algorithmic management and innovation performance, with job crafting playing a subsequent mediating role in both pathways. Notably, the indirect effects involving perceived fit (*β* = 0.125 and 0.115) were substantially larger than those involving perceived control (*β* = 0.036 and 0.020), suggesting that the resource-driven pathway (perceived fit) is a stronger mechanism than the demand-consumption pathway (perceived control).

## 5 Discussion

### 5.1 The effects of algorithmic management on perceived fit and perceived control

According to the empirical analysis, the effect of algorithmic management on perceived fit (*β* = 0.512, *p* < 0.001) is substantially larger than its effect on perceived control (*β* = 0.269, *p* < 0.001). This indicates that, in the context of high-technology enterprises, the “resource attribute” of algorithmic management is more readily perceived and accepted by employees than its “demand attribute.”

This finding is consistent with Keegan and Meijerink (2025) and Liang et al. (2025), who emphasized the positive role of algorithmic management in enhancing employees perceived fit. When algorithmic systems optimize task allocation and provide clear performance feedback based on employees’ skills, preferences, and needs, employees perceive a strong alignment between themselves and their work tasks, thereby interpreting algorithmic structures as supportive resources.

In the context of high-technology enterprises, this may be because employees in such settings generally possess higher levels of professional autonomy and cognitive ability, making them more inclined to view algorithmic systems as tools for improving work efficiency rather than as mechanisms that constrain their behavior. This conclusion offers important implications for enterprises implementing algorithmic management systems: organizations should focus on leveraging the advantages of algorithmic management in task matching and performance feedback.

### 5.2 The mediating role of perceived fit and perceived control

The study found that the mediating effect of perceived fit (*β* = 0.125) was 3.5 times larger than that of perceived control (*β* = 0.036). This difference suggests that, in the context of high-technology enterprises, the “resource-driven mechanism” of algorithmic management is substantially stronger than its “demand-consumption mechanism.”

A possible explanation is that employees in high-technology enterprises generally possess higher levels of professional autonomy and cognitive ability, which may attenuate the negative impact of perceived control on innovation performance. Additionally, the real-time feedback and transparent rules provided by algorithmic systems may be recognized and utilized by employees before feelings of control take hold, thereby reinforcing the salience of perceived fit.

These findings are partially consistent with Zhu et al. (2024) and Zhu et al. (2025), who found that the impact of perceived control on work outcomes involves both positive and negative pathways, depending on how employees interpret and respond to algorithmic control. The present findings further enrich this perspective by demonstrating that, in the context of high-technology enterprises, the negative effects of perceived control are significantly weakened, while the positive effects of perceived fit are strengthened.

### 5.3 The chain mediating roles of perceived fit, perceived control, and job crafting

The study found that the chain mediating effect through perceived fit and job crafting (*β* = 0.115) was 5.75 times larger than that through perceived control and job crafting (*β* = 0.020). These findings further validate the Job Demands-Resources theory and Self-Determination Theory. They are consistent with Liu and Yin (2024) and Zhu et al. (2025), and extend prior research by demonstrating that, in the context of high-technology enterprises, the “resource-driven chain mechanism” (AIM → PF → JC → IP) is substantially stronger than the “demand-consumption chain mechanism” (AIM → PC → JC → IP), with the positive chain mechanism dominating in high-technology enterprise contexts.

In contrast, although perceived control promotes job crafting to some extent, its effect is relatively weak. This is because employees need to expend psychological energy coping with continuous monitoring and rule constraints, leaving insufficient psychological resources to support proactive job crafting behaviors. This finding further supports the core proposition of the Job Demands-Resources theory: when job demands are too high, employees’ resources become excessively depleted, and proactive behaviors are weakened.

## 6 Conclusion

Drawing on the Job Demands-Resources theory and Self-Determination Theory, this study explored the mechanisms through which algorithmic management affects employee innovation performance in high-technology enterprises. The main conclusions are as follows.

First, the “resource attribute” of algorithmic management is stronger than its “demand attribute.” The empirical results show that the effect of algorithmic management on perceived fit (*β* = 0.512) is substantially larger than its effect on perceived control (*β* = 0.279). This indicates that, in the context of high-technology enterprises, employees primarily experience algorithmic systems as a supportive resource (enhancing task matching and efficiency) rather than a constraining control mechanism. This finding refines the understanding of the double-edged sword effect of algorithmic management, revealing the dominance of its positive effects in high-technology enterprise contexts.

Second, perceived fit is the core psychological mechanism through which algorithmic management promotes innovation. The mediating effect of perceived fit (*β* = 0.125) is 3.5 times larger than that of perceived control (*β* = 0.036). Moreover, the chain mediating effect of perceived fit and job crafting (*β* = 0.115) is 5.75 times larger than that of perceived control and job crafting (*β* = 0.020). This confirms that the “resource-driven chain mechanism” is the dominant pathway through which algorithmic management influences employee innovation, while the role of the “demand-consumption mechanism” is relatively limited.

Based on the above findings, this study proposes the following three practical implications. First, leverage the resource attribute of algorithmic management to enhance employees perceived fit. Organizations should focus on utilizing the advantages of algorithmic systems in task allocation and performance feedback, optimizing task matching based on employees’ skills, preferences, and needs, and providing timely and transparent performance feedback. This helps enhance employees’ sense of control and fit with work tasks, thereby stimulating their intrinsic motivation and work engagement.

Second, avoid excessive control and maintain algorithmic flexibility and adaptability. Although the negative effects of perceived control are relatively weak in high-technology enterprise contexts, organizations should still be cautious about the potential risks of excessive control. It is recommended that organizations preserve a certain degree of employee autonomy when designing algorithmic systems, allowing employees to make moderate work adjustments and innovative attempts within the algorithmic framework.

Third, promote employee innovation through job crafting. Organizations should encourage employees to engage in job crafting behaviors, such as adjusting task execution methods, seeking additional resources, and reframing work meaning. Through training, empowerment, and incentive mechanisms, organizations can cultivate employees’ awareness and ability to craft their jobs, thereby maximizing the positive effects of algorithmic management.

Although this study has yielded valuable conclusions, it still has certain limitations. This study employed cross-sectional data, making it difficult to fully establish causal relationships among the variables. Future research should adopt longitudinal designs or experimental methods to further validate the findings. In addition, the sample was drawn exclusively from high-technology enterprises, and the generalizability of the conclusions to other industries and cultural contexts needs to be further tested. Future research should also explore the boundary effects of moderating variables such as employee personality traits on the main effects and mediating effects.

## Data Availability

The raw data supporting the conclusions of this article will be made available by the authors, without undue reservation.
